# Primary Biliary Cholangitis and Chronic Arterial Hypertension in Pregnancy

**DOI:** 10.7759/cureus.70453

**Published:** 2024-09-29

**Authors:** Mariana O Santos, Inês Marques, Carlos Barata, Maria Céu Almeida

**Affiliations:** 1 Department of Obstetrics and Gynecology, Unidade Local de Saúde (ULS) Coimbra, Coimbra, PRT; 2 Department of Obstetrics, Maternidade Bissaya Barreto, Unidade Local de Saúde (ULS) Coimbra, Coimbra, PRT

**Keywords:** case report, hypertension, preeclampsia, pregnancy, primary biliary cholangitis

## Abstract

This case report presents a woman with a history of adverse obstetric outcomes: two pregnancies complicated by preeclampsia, resulting in a medical termination in the first and fetal demise in the second. Prior to a subsequent pregnancy, she underwent investigations for hypertension, thrombophilia, and autoimmune diseases. These investigations led to a probable diagnosis of primary biliary cholangitis (PBC) and chronic hypertension (CH). She was medicated for both conditions and closely monitored at Unidade Local de Saúde (ULS) Coimbra. Due to severe preeclampsia, she underwent an emergency cesarean section at 33 weeks, delivering a live male infant weighing 1635 g.

## Introduction

Maternal hypertension is a leading cause of maternal and perinatal mortality. Preeclampsia, which complicates between 5% and 7% of all pregnancies, is a progressive, multisystem disorder characterized by the onset of hypertension and proteinuria or hypertension and organ dysfunction after 20 weeks of gestation [1]. Primary biliary cholangitis (PBC) is a rare, chronic, immune-mediated cholestatic liver disease characterized by inflammation of the bile ducts, which can lead to cirrhosis and liver failure. This disease is more prevalent in women.

## Case presentation

A 42-year-old woman was referred to the Preconceptional Consultation at Bissaya Barreto Maternity Hospital (ULS Coimbra) due to two previous pregnancies complicated by preeclampsia (PE). The first pregnancy resulted in a medical termination at 21 weeks, and the second in a fetal demise at 25 weeks. Both pregnancies were conceived through in vitro fertilization with donor gametes.

As part of this consultation, she underwent a hypertension study using ambulatory blood pressure monitoring (ABPM), which revealed chronic hypertension; an electrocardiogram and echocardiogram showed no abnormalities; and a renal ultrasound was also unremarkable. She was then started on nifedipine 30 mg daily.

She had previously been diagnosed with autoimmune thyroiditis and had been regularly monitored for thyroid function during pregnancy, with levothyroxine doses adjusted accordingly.

Thrombophilia testing was unremarkable, but autoimmune disease testing revealed the presence of anti-pyruvate DH IgG-M2 and anti-pyruvate DH (immunoglobulin (Ig)G, IgA, IgM) - M2 antibodies. She was referred to the Autoimmune Diseases Clinic in the Internal Medicine department at ULS Coimbra, and a probable diagnosis of primary biliary cholangitis (PBC) was made. She was started on ursodeoxycholic acid 250 mg twice daily and advised to consider the risks of another pregnancy.

She underwent assisted reproductive technology again and became pregnant via frozen embryo transfer (with donor gametes). During pregnancy, she was treated with nifedipine 30 mg daily, aspirin 100 mg, enoxaparin 40 mg, and ursodeoxycholic acid 250 mg twice daily.

**Figure 1:**
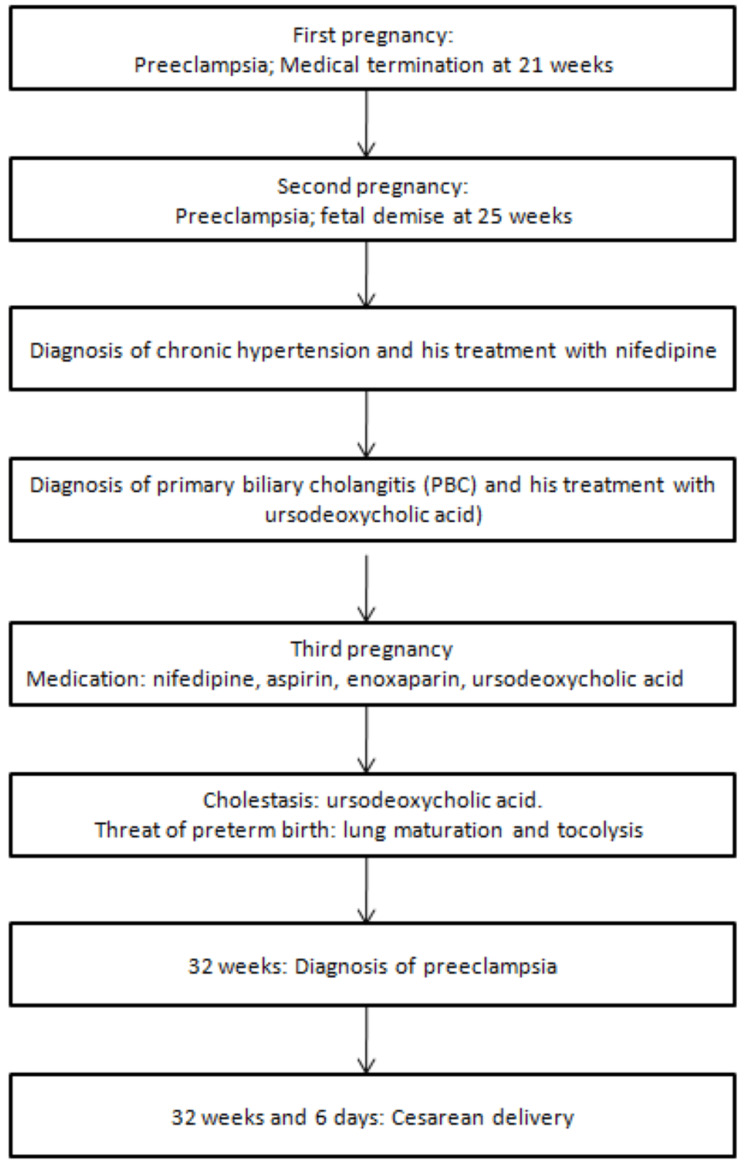
Timeline of clinical events

Her blood pressure remained normal until 29 weeks, when she was admitted to the Obstetrics department of Bissaya Barreto Maternity Hospital for therapeutic adjustment. She received corticosteroid therapy and increased her nifedipine dose to 30 mg twice daily. At 32 weeks of gestation, she was readmitted with a diagnosis of preeclampsia. She underwent another course of fetal lung maturity testing. At 32 weeks and 6 days, she delivered a male infant by cesarean section, weighing 1635g with Apgar scores of 9/10/10 (Figure 1).

## Discussion

Hypertension, defined as a systolic blood pressure of ≥ 140 mmHg and diastolic blood pressure of ≥ 90 mmHg, is a common complication of pregnancy. Chronic hypertension is defined as hypertension that occurs before conception or before 20 weeks of gestation. Its incidence has been increasing and is present in 0.9-1.5% of pregnant women [2]. Hypertension can lead to maternal problems, such as preeclampsia, target organ dysfunction (renal failure, acute myocardial infarction, pulmonary edema, and death); and fetal problems such as fetal growth restriction, preterm birth, and fetal/neonatal death.

The use of appropriate antihypertensive medications, such as labetalol (a beta-blocker with alpha-blocking action) and nifedipine (a calcium channel blocker), can improve pregnancy outcomes [3].

Preeclampsia is a systemic disorder that affects approximately 10 million pregnancies per year and increases the risk of maternal and fetal morbidity and mortality [1]. The pathogenesis of preeclampsia involves changes in placentation and systemic maternal vascular changes. Most cases occur in the late preterm phase (34-37 weeks), term, and postpartum, with better maternal and fetal outcomes than when it occurs earlier in the preterm period [4].

The treatment of preeclampsia is delivery. The use of low-dose aspirin reduces the risk of preeclampsia [5]. Aspirin decreases the synthesis of platelet thromboxane and maintains the synthesis of prostacyclin [6]. Thromboxane has the opposite effect of prostacyclin, promoting platelet aggregation and vasoconstriction [6]. Administration of this drug can thus reduce the risk of preeclampsia and its consequences in high-risk patients [7].

Primary biliary cholangitis (PBC) is a rare, autoimmune liver disease. The pathogenesis of the disease is multifactorial and not fully understood [8]. It is believed to be mediated by T lymphocytes, which cause continuous alterations in the bile duct epithelium, leading to its gradual destruction, with the influence of genetic, environmental, and gut-liver axis factors. The alterations caused in the bile ducts can lead to their disappearance and, ultimately, cirrhosis and liver failure.

Patients with PBC are often diagnosed with other autoimmune diseases and may be asymptomatic or present with symptoms such as fatigue and pruritus. The presence of antimitochondrial antibodies (AMA) at a titer greater than 1:140 or more and alterations in liver tests with an alkaline phosphatase increased by one and a half times the upper normal limit make the diagnosis, eliminating the need for liver biopsy. However, a biopsy can also allow for a diagnosis. AMA antibodies are present in almost all patients and are considered the serological markers of PBC. There are four main autoantigens, known globally as "M2" (the E2 subunits of the pyruvate dehydrogenase complex), the branched-chain 2-oxo-acid dehydrogenase complex, the ketoglutarate dehydrogenase complex, and the dihydrolipoamide dehydrogenase binding protein [3]. Ursodeoxycholic acid is used to treat the progression of the disease.

## Conclusions

Early identification, treatment, and management of chronic conditions in women planning pregnancy often lead to successful pregnancy outcomes. A multidisciplinary team plays a crucial role in this process. By addressing underlying health issues before conception, potential complications, such as preeclampsia, preterm birth, and fetal growth restriction, can be minimized. In this case, although the patient had multiple risk factors, a comprehensive approach involving close monitoring and tailored medical therapy allowed for a relatively favorable outcome.
